# Supplementary material for: From sequence to enzyme mechanism using multi-label machine learning
Source: BMC Bioinformatics. 2014 May 19;15:150. doi: 10.1186/1471-2105-15-150 (PMC4229970; doi:10.1186/1471-2105-15-150)
Supplement: Additional file 2 — Java code of ml2db. Additional file ml2db_code.tar.gz contains the Java source code to run the multi-label machine learning experiments and save the results to database. The code’s Javadoc is included. [file 1471-2105-15-150-S2.zip › additional file 2/ml2db/ml2db/doc/main/ml2db/weka/JaccardDistance.html]

JaccardDistance


JavaScript is disabled on your browser.


- Overview
- Package
- Class
- Use
- Tree
- Deprecated
- Index
- Help

- Prev Class
- Next Class

- Frames
- No Frames

- All Classes

- Summary:
- Nested |
- Field |
- Constr |
- Method

- Detail:
- Field |
- Constr |
- Method


main.ml2db.weka

## Class JaccardDistance

- java.lang.Object
- - weka.core.NormalizableDistance
  - - main.ml2db.weka.JaccardDistance

- All Implemented Interfaces:
  :   java.io.Serializable, weka.core.DistanceFunction, weka.core.OptionHandler, weka.core.RevisionHandler

  Direct Known Subclasses:
  :   JaccardEuclideanDistance

  ---

    

  ```
  public class JaccardDistance
  extends weka.core.NormalizableDistance
  implements java.io.Serializable
  ```

  This is an implementation of the Jaccard distance which calculates it on the
  fly. The Jaccard distance = 1 minus the Jaccard coefficient. The Jaccard
  coefficient measures similarity between sample sets, and is defined as the
  size of the intersection divided by the size of the union of the sample sets.

  Version:
  :   22 Apr 2013

  Author:
  :   ldf

  See Also:
  :   Serialized Form

- - ### Field Summary

    - ### Fields inherited from class weka.core.NormalizableDistance

      `R_MAX, R_MIN, R_WIDTH`
  - ### Constructor Summary

    Constructors

    Constructor and Description

    `JaccardDistance()`

    `JaccardDistance(weka.core.Instances data)`
  - ### Method Summary

    Methods

    | Modifier and Type | Method and Description |
    | --- | --- |
    | `double` | `distance(weka.core.Instance a, weka.core.Instance b)` |
    | `double` | `distance(weka.core.Instance first, weka.core.Instance second, double cutOffValue)` |
    | `double` | `distance(weka.core.Instance first, weka.core.Instance second, double cutOffValue, weka.core.neighboursearch.PerformanceStats stats)` |
    | `int` | `getIntersection(java.util.TreeSet<java.lang.Integer> a, java.util.TreeSet<java.lang.Integer> b)` Calculate the size of the intersection of set A (feature attributes of instance A) and set B (feature attributes of instance B), that is, the non-zero attributes they have in common. |
    | `java.lang.String` | `getRevision()` |
    | `int` | `getUnion(java.util.TreeSet<java.lang.Integer> a, java.util.TreeSet<java.lang.Integer> b)` Calculate the size of the union of set A (feature attributes of instance A) and set B (feature attributes of instance B), that is, all the non-zero attributes the two instances have. |
    | `java.lang.String` | `globalInfo()` |

    - ### Methods inherited from class weka.core.NormalizableDistance

      `attributeIndicesTipText, distance, dontNormalizeTipText, getAttributeIndices, getDontNormalize, getInstances, getInvertSelection, getOptions, getRanges, initializeRanges, initializeRanges, initializeRanges, initializeRangesEmpty, inRanges, invertSelectionTipText, listOptions, postProcessDistances, rangesSet, setAttributeIndices, setDontNormalize, setInstances, setInvertSelection, setOptions, toString, update, updateRanges, updateRanges, updateRanges, updateRangesFirst`
    - ### Methods inherited from class java.lang.Object

      `equals, getClass, hashCode, notify, notifyAll, wait, wait, wait`

- - ### Constructor Detail


    - #### JaccardDistance

      ```
      public JaccardDistance()
      ```


    - #### JaccardDistance

      ```
      public JaccardDistance(weka.core.Instances data)
      ```
  - ### Method Detail


    - #### distance

      ```
      public double distance(weka.core.Instance a,
                    weka.core.Instance b)
      ```

      **Specified by:**
      :   `distance` in interface `weka.core.DistanceFunction`

      **Overrides:**
      :   `distance` in class `weka.core.NormalizableDistance`


    - #### distance

      ```
      public double distance(weka.core.Instance first,
                    weka.core.Instance second,
                    double cutOffValue)
      ```

      **Specified by:**
      :   `distance` in interface `weka.core.DistanceFunction`

      **Overrides:**
      :   `distance` in class `weka.core.NormalizableDistance`


    - #### distance

      ```
      public double distance(weka.core.Instance first,
                    weka.core.Instance second,
                    double cutOffValue,
                    weka.core.neighboursearch.PerformanceStats stats)
      ```

      **Specified by:**
      :   `distance` in interface `weka.core.DistanceFunction`

      **Overrides:**
      :   `distance` in class `weka.core.NormalizableDistance`


    - #### getIntersection

      ```
      public int getIntersection(java.util.TreeSet<java.lang.Integer> a,
                        java.util.TreeSet<java.lang.Integer> b)
      ```

      Calculate the size of the intersection of set A (feature attributes of
      instance A) and set B (feature attributes of instance B), that is, the
      non-zero attributes they have in common.

      Parameters:
      :   `a` -
      :   `b` -

      Returns:
      :   the size of the intersection of set A (feature attributes of
          instance A) and set B (feature attributes of instance B), that
          is, the non-zero attributes they have in common.


    - #### getRevision

      ```
      public java.lang.String getRevision()
      ```

      **Specified by:**
      :   `getRevision` in interface `weka.core.RevisionHandler`


    - #### getUnion

      ```
      public int getUnion(java.util.TreeSet<java.lang.Integer> a,
                 java.util.TreeSet<java.lang.Integer> b)
      ```

      Calculate the size of the union of set A (feature attributes of instance
      A) and set B (feature attributes of instance B), that is, all the
      non-zero attributes the two instances have.

      Parameters:
      :   `a` -
      :   `b` -

      Returns:
      :   the size of the union of set A (feature attributes of instance A)
          and set B (feature attributes of instance B)


    - #### globalInfo

      ```
      public java.lang.String globalInfo()
      ```

      **Specified by:**
      :   `globalInfo` in class `weka.core.NormalizableDistance`


- Overview
- Package
- Class
- Use
- Tree
- Deprecated
- Index
- Help

- Prev Class
- Next Class

- Frames
- No Frames

- All Classes

- Summary:
- Nested |
- Field |
- Constr |
- Method

- Detail:
- Field |
- Constr |
- Method
